# Recent Advances in Flexible RF MEMS

**DOI:** 10.3390/mi13071088

**Published:** 2022-07-08

**Authors:** Yingli Shi, Zhigang Shen

**Affiliations:** 1 School of Materials and Energy, University of Electronic Science and Technology of China (USETC), Chengdu 610054, China; yingli.shi@uestc.edu.cn; 2 Beijing Key Laboratory for Powder Technology Research and Development, Beihang University (BUAA), Beijing 100191, China

**Keywords:** RF MEMS, flexible substrate, bending deformation, RF characteristics, reconfigurable

## Abstract

Microelectromechanical systems (MEMS) that are based on flexible substrates are widely used in flexible, reconfigurable radio frequency (RF) systems, such as RF MEMS switches, phase shifters, reconfigurable antennas, phased array antennas and resonators, etc. When attempting to accommodate flexible deformation with the movable structures of MEMS, flexible RF MEMS are far more difficult to structurally design and fabricate than rigid MEMS devices or other types of flexible electronics. In this review, we survey flexible RF MEMS with different functions, their flexible film materials and their fabrication process technologies. In addition, a fabrication process for reconfigurable three-dimensional (3D) RF devices based on mechanically guided assembly is introduced. The review is very helpful to understand the overall advances in flexible RF MEMS, and serves the purpose of providing a reference source for innovative researchers working in this field.

## 1. Introduction

**Figure 1:**
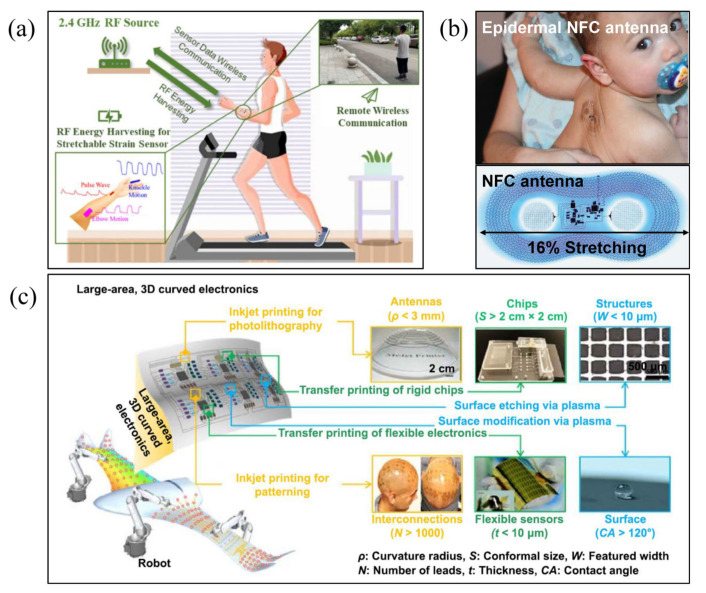
Application requirements of reconfigurable, flexible RF devices in flexible conformal electronics. (**a**) The concept of a scalable RF system is to communicate wirelessly with the body [10], Reprinted/adapted with permission from Ref. [10]. 2022, Elsevier. (**b**) An NFC antenna for neonatal monitoring, which can be attached to areas of large skin deformation that result from wrinkling [11]. (**c**) Large area, multi-functional Flexible Smart Skin with conformal antennas and integrated sensors [12].

Driven by the exponential growth in wireless data usage around the globe [1], wireless communication technologies and network capacity are advancing at unprecedented speeds. For consumer communication electronics with high integration and miniaturization, there is an urgent demand for lightweight and reconfigurable communication component systems that can arrange antennas and other RF-integrated circuits within them in order to cover more frequency bands in a limited compact space [2]. Based on common design methods, there is almost no spare space for RF components that are compatible with more frequency bands for the latest 5G (fifth-generation) mobile smartphones using highly integrated antennas [3]; however, the miniaturized, flexible, reconfigurable RF technology provides a promising solution to this challenge [2,4]. The concept of flexible electronics that has the advantages of being flexible, miniaturized and highly integrated is a charming technology, which has unique technical advantages in application scenarios such as bio-integrated electronics and complex structural surface conformal integration. Whether applied to signal monitoring in biological systems [5,6,7], or in conformal applications [8,9], integrated systems for flexible electronics can sense and collect massive amounts of information. The real-time transmission of conformal information in these systems places high demands on flexible wireless transmission systems. Miniaturized, highly integrated, flexible RF components act as an essential link between the signal sensing and external wireless communication systems described above, in order to satisfy the demands of multi-band integrated transmission; such flexible RF systems are generally reconfigurable. Figure 1a,b demonstrate the concept of a scalable RF system used to communicate wirelessly with the body [10], and an epidermal near field communication (NFC) antenna for neonatal monitoring that can be attached to areas of large skin deformations that result from wrinkling [11], respectively. As a typical complex, curved structure monitoring application, large area, multi-functional Flexible Smart Skins with conformal antennas and integrated sensors [12] are shown in Figure 1c.

RF MEMS refers to RF devices and systems manufactured for communication and information processing that are based on MEMS processing technology. Compared with traditional RF microelectronic devices, RF MEMS have the following advantages: miniaturization, light weight, low insertion loss, high isolation, short response time, strong integration capabilities, etc. These excellent characteristics are particularly suitable for the use of high integration and reconfigurable application scenarios [13]. MEMS devices based on flexible substrates are widely used in flexible RF systems and their reconfigurable systems, such as flexible RF MEMS switches, phase shifters, reconfigurable antennas, phased array antennas, flexible resonators, etc. Despite their excellent performance and wide application, considering the compatibility of flexible deformation with microsystems with movable structures, flexible RF MEMS are far more difficult to structurally design and fabricate than rigid MEMS devices or other types of flexible electronics. In order to deepen the research and understanding of flexible RF MEMS, it is necessary to sort out their functions, structural design, fabrication processes, materials and characteristics, etc.

Table 1 shows a comparison of recent literature reviews on RF MEMS, or flexible MEMS. It can be seen that there are many reviews on RF MEMS which cover their research progress in various application fields; however, these RF MEMS are basically rigid. As for flexible MEMS, most of the existing reviews focus on flexible MEMS actuators, sensors, fabrication processes and materials, etc., and there are no reviews on the progress of flexible MEMS in RF applications. Therefore, a comprehensive summary of the research progress of flexible RF MEMS is of great significance, in order to fill in the gaps that exist in this research field.

The purpose of this review is to provide valuable references for the structural designs and performance improvements in flexible RF MEMS based on the achievements of the past 30 years. In the Section 2, the research progress of flexible RF MEMS with different functions, mainly in switches, phase shifters, reconfigurable and phased array antennas and resonators, is discussed. The third part introduces the flexible film materials used in flexible RF MEMS, including polymer materials, thinned rigid substrates and structural materials. Reasonable structural design and material selection can ensure the integrity of the structure and function. The Section 4 arranges the sorts of processing means for flexible RF MEMS, including modified surface MEMS processing, the transfer printing fabrication method of flexible electronics and printing technology based on additive manufacturing. In the Section 6 of this paper, the flexible reconfigurable 3D RF devices based on mechanical self-assembly and mechanically triggered switches are discussed. Flexible deformation is not only a factor that limits the performance of such flexible 3D RF devices; it is also applied to the entire process of device fabrication and operation, which is a different development strategy from the aforementioned flexible RF MEMS. The development trends in flexible RF-MEMS are prospected. This paper aims to provide reference for the research conducted in this field.

## 2. Flexible RF MEMS with Different Functions

At present, some progress has been made in the research of various functional flexible RF MEMS, mainly in flexible RF MEMS switches [46,47,48,49,50,51,52,53,54,55,56,57,58,59,60,61,62,63,64,65,66,67,68,69,70,71,72,73,74,75], phase shifters [76,77,78], reconfigurable antennas [78,79,80,81,82,83,84,85,86] and phased array antennas [87,88,89,90] based on them, in MEMS resonators [91,92,93,94,95,96,97,98,99,100,101,102,103], frequency selective hyper surfaces [104], etc.

### 2.1. Flexible RF MEMS Switches

As the basic unit of flexible RF MEMS, research in switches has received the most attention. In order to understand the research progress related to flexible MEMS switches more clearly and intuitively, the technical summary of flexible RF MEMS switches is given in Table 2, mainly including substrate materials, structural materials, structural characteristics, manufacturing processes, etc.

As early as 2001, a reconfigurable conformal antenna based on MEMS and flexible membranes has been proposed for the application of space-based radar [46]. Using advanced MEMS and packaging technology, the antenna and the electronics under the ground plane can be folded or rolled together with the flexible substrate. The reconfigurable antenna based on MEMS switching can switch between two broadbands, S-band and X-band, at a driving voltage of less than 10 V. Although this achievement does not use a fully flexible MEMS structure, it creates a precedent for the research and application of flexible RF MEMS. 

**Figure 2:**
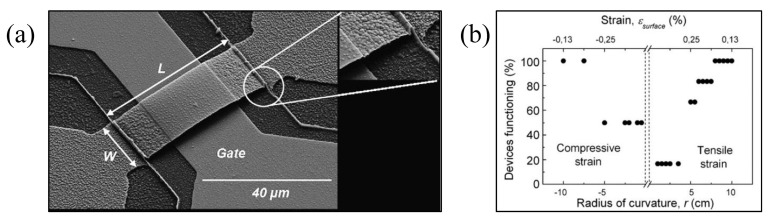
(**a**) Thin-film silicon beam on a PET film [48] Reprinted/adapted with permission from Ref. [48]. 2008, Elsevier. (**b**) Influence of substrate bending on device function [48] Reprinted/adapted with permission from Ref. [48]. 2008, Elsevier.

Wang et al. [47] developed a cantilever membrane beam RF MEMS switch that was supported by two fixed-free beams integrated on a liquid crystal polymer (LCP) flexible substrate. Wang et al. [49] developed an air-bridge coplanar waveguide (CPW) switch based on LCP, which can integrate 3D RF front-end reconfigurable architecture on LCP. The research also shows that LCP-based switches can be used as a low-cost or enhanced performance alternative to silicon-based RF MEMS switches. For this LCP switch, the actual measured pull-down voltage is 25 V. When the switch is active, the isolation is about 20 dB at 20 GHz, while the corresponding return loss is about 0.1 dB. When the switch is in the UP position, the insertion loss is about 0.08 dB at 20 GHz, while the corresponding return loss is 18 dB. These findings initiated the study of flexible RF MEMS. Patil et al. [48] fabricated thin film MEMS micro-beams on a 250-micrometer-thick flexible polyethylene terephthalate (PET) film; their properties in the manufacturing state and after substrate bending were evaluated, as shown in Figure 2a,b. Bending states of the substrate show that 16.6% of the devices can withstand 1.25% tensile strains, while a larger number of devices, 50%, can withstand a higher compressive strain of −2.5%. Thin-film silicon beams prepared on polyethylene terephthalate (PET) substrates demonstrate resonance frequencies of up to 2 MHz and quality factors values of about 500 when measured in a vacuum.

**Figure 3:**
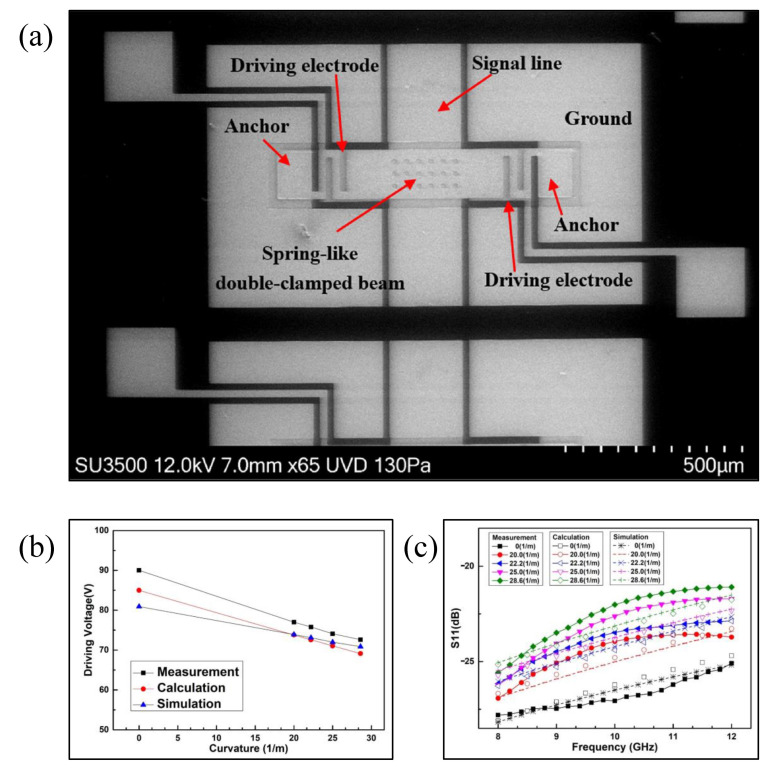
(**a**) Spring-like beam electrostatically actuated flexible RF MEMS switch [67]. (**b**) Measured driving time [67] and (**c**) reflection loss S11 [67] of the switch measured under different curvatures.

Han et al. [57,66,67] proposed an RF MEMS switch fabricated onto a flexible LCP substrate, and studied the influence of substrate bending on the mechanical properties of the switch; a spring-like double-clamped CPW switch was fabricated, as shown in Figure 3a. For the designed flexible switch, a substrate bending modeling method is proposed in this study. Theoretical analysis is carried out in combination with simulation and measurement. Han et al. [67] proposed multi-physical characteristics models based on substrate bending, including the static structural, dynamic structural and microwave characteristic models of double-ended fixed beam switches that are based on substrate flexible deformation. Through simulation and experiment, it was revealed that the driving voltage and response time of the switch are inversely proportional to the increase in bending curvature of the substrate. With an increase in substrate curvature, the microwave performance of the switch becomes worse in the ON state. The switch driving time and reflection loss S11 measured under different curvatures are shown in Figure 3b,c [67]. Han et al. [69] proposed a flexible V-shaped beam thermal actuator switch, and analyzed its driving characteristics under bending conditions.

**Figure 4:**
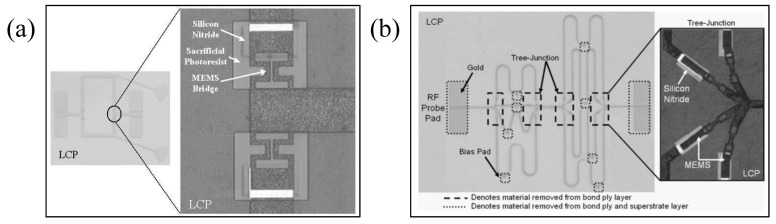
(**a**) Fabricated MEMS switch and phase shifter based on LCP substrate used in the packaging process [77] Reprinted/adapted with permission from Ref. [77]. 2005, IEEE. (**b**) Miniaturized, low-loss 4-bit flexible RF MEMS phase shifter [78] Reprinted/adapted with permission from Ref. [78]. 2006, IEEE.

John and his team [55,56,57] designed and fabricated a flexible MEMS switch with packaging, and characterized the moisture and water resistance characteristics of the switch with its packaging structure. The sealing quality was tested by submerging the encapsulated switch in 60 °C water for 24 h. The measurement after soaking indicated that the bonding technique was successful. The fabricated switch used in the packaging process is shown in Figure 4a [77]. The flexible RF MEMS switch encapsulated in LCP substrate is able to function normally for 30 min at 85 °C and at 85% relative humidity, indicating that this packaging technique can withstand a certain degree of moisture exposure.

Takao and his team [53,71,72,73] combined the advanced printing technology and flexible MEMS technology to develop a large-area wireless power delivery system on polyimide (PI) films. Complementary circuits that integrate flexible MEMS switches and organic transistors make large-area, flexible and high-power-density applications realized. Since all components of this wireless transmission system are made on plastic film, it is convenient to put the wireless power delivery board on tables, floors, walls and many other possible locations. 

### 2.2. Flexible RF MEMS Phase Shifter

The RF MEMS phase shifter is an integral component in radar and communication systems. Recently, flexible phase shifters based on MEMS switching have made some research achievements. John’s team [77] proposed the use of an MEMS phase shifter on flexible substrate for the first time. In order to apply the bias voltage that drives the MEMS switch, a radial stub is designed on each of the two signal lines, as shown in Figure 4a [77]. The measurement results of a two-position MEMS phase shifter show decent performance. At the design frequency, the return loss is about 22.5 dB, while the insertion loss is about 0.98 dB. The phase error is 1.26°, centered on the design frequency. 

On the basis of this research, John’s team [78] proposed a miniaturized, low-loss 4-bit phase shifter, which was integrated into an all organic package, as shown in Figure 4b [78]. Furthermore, John’s team [76] presented a miniaturized, low-voltage X-band phase shifter integrated in a multi-layer organic package with a single-pole four-throw (SP4T) MEMS switch. The multi-layer LCP fabrication processing technology is compatible with low-cost and lightweight circuits, and can easily be integrated and packaged with other types of RF front-end components, such as antennas.

As an important component of reconfigurable RF systems, phase shifters are often used to integrate with antennas. Nickolas et al. [80] proposed a reconfigurable RF MEMS phased array antenna that was integrated into an LCP system package, which consisted of an LNA, MEMS phase shifter and patch antenna array. The RF MEMS phase shifter used here is a pair of single-bit phase shifters that were fabricated on an LCP substrate in order to provide beam steering for the antenna.

### 2.3. Flexible RF MEMS Antenna

**Figure 5:**
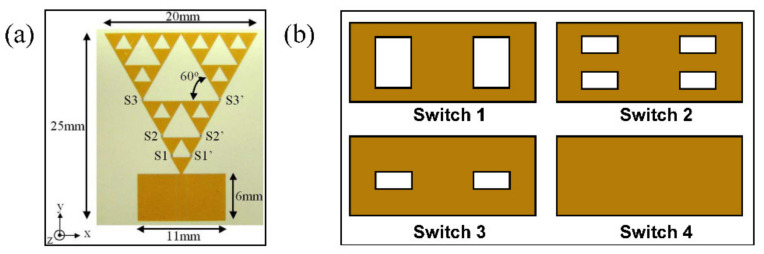
(**a**) Photograph of the fabricated reconfigurable antenna with MEMS switches [83] Reprinted/adapted with permission from Ref. [83]. 2007, IEEE. (**b**) Varieties of fabricated MEMS beams.

Due to the outstanding “off” state isolation of MEMS switches, the placement of additional switches has little effect on these devices. This technology is well suited for devices or systems that require significant reconfigurability; moreover, they can withstand small increases in size that come with add-on switches, such as reconfigurable antennas. Nickolas et al. [83] presented a CPW-fed gasket monopole antenna with a three-order iterative Sierpinski structure to reconstruct the antenna by controlling the different MEMS switch states, as shown in Figure 5a [83] and Figure 5b. Different parts of the reconfigurable antenna geometry are activated and disabled sequentially by adjusting the direct current (DC) voltage at the RF feed. This design avoids setting a DC bias line at each MEMS switch, simplifies the antenna structure and improves the RF characteristics of the antenna. The reconfigurable device does not employ any additional bias circuit to actuate the switches, and the antenna has excellent radiation characteristics of four main resonant frequencies. Such a design based on MEMS switches can reduce losses in the antenna.

Nickolas et al. [80] proposed an RF MEMS-integrated phased antenna based on a flexible organic substrate. The integrated phased antenna system is mainly composed of a patch antenna array, RF power distribution network, phase shifter and low noise amplifier (LNA). Patch antennas are used to receive radiation signals. LNAs provide signal amplification function. RF MEMS phase shifters based on LCP control the phase of microwave signals. When the LCP substrate thickness is 100 μm, the loss on the transmission line is 0.375 dB/cm. The total length of the feedback network is 10.04 cm and the total loss is 3.77 dB, of which 0.34 dB is from the phase shifter. Each switch yields an additional 0.2 dB loss. The design uses LCP material as the substrate plate and packaging material, which helps this system achieve miniaturization, low cost, low losses and high flexibility, and enables it to meet the customization of all sizes of equipment. Wang et al. [79] presented an antenna array with dual frequencies and dual polarizations based on multi-layer LCP technology. Two 2 × 2 arrays operating at 14 and 35 GHz have been designed. These antenna arrays can be integrated with 3D RF components, including integrated circuits, filters and embedded passives to achieve a complete RF front end system all contained in a single package.

There are also some flexible reconfigurable antennas based on MEMS switches that are applied to various conformal application scenarios, such as reconfigurable and conformal patch antennas [82], reconfigurable and conformal patch antennas for body wireless communications [84], reconfigurable antennas based on RF switches in satellite communications [85], and reconfigurable antennas on flexible substrates for X-band [86], flexible lightweight multi-layer 16 × 16 antenna arrays [89]. However, these reconfigurable conformal antennas are not completely flexible systems, in which the MEMS switch is integrated with a rigid substrate; however, thanks to the small size of the switch, it has little impact on the flexible application of the entire antenna array.

### 2.4. Flexible RF MEMS Resonator

Micro resonators are widely used MEMS devices in RF filtering, timing, motion sensing, stress sensing, chemical sensing and other fields. The mature process of micro resonators is based on silicon substrates, but in recent years the fabrication process and applications of micro resonators based on glass and polymer substrates have attracted growing attention.

**Figure 6:**
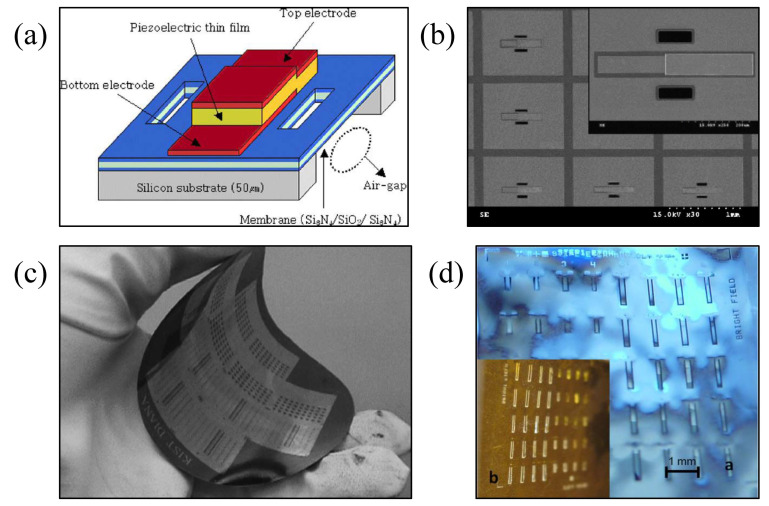
(**a**) Structure and (**b**) SEM of an FBAR based on an ultra-thin flexible silicon substrate [93] Reprinted/adapted with permission from Ref. [93]. 2005, Elsevier. (**c**) Fabricated thin FBAR of wafer level with flexibility [93] Reprinted/adapted with permission from Ref. [93]. 2005, Elsevier. (**d**) MEMS resonant cantilevers based on flexible PI film [92] Reprinted/adapted with permission from Ref. [92]. 2012, Elsevier.

Kang et al. [93] proposed a thin-film bulk acoustic resonator (FBAR) structure with cantilever beam based on an ultra-thin flexible silicon wafer; its structure and scanning electron microscope (SEM) images are shown in Figure 6a,b [93]. In this structure, the substrate is made flexible by reducing the thickness of silicon wafer to 50 μm, as shown in Figure 6c [93], and then the membrane beam structure is realized by using a reactive ion etching (RIE) process. The device is fabricated via the standard MEMS process, with high machining accuracy and good device performance. However, the thinned silicon wafer can only withstand a small amount of bending deformation with its poor ductility. Petroni et al. [92] first proposed the preparation of a flexible micro cantilever that uses aluminum nitride (AlN) as a piezoelectric (PZT) thin active film based on a flexible polyimide (PI) substrate. Due to the residual stress of the multi-layer metal sandwich structure on the PI, the flexible cantilever is bent downward. The flexible cantilever mechanical behavior is studied using piezoelectric response measurement. The first resonance frequency obtained from the experiment is about 15 kHz. Pestana et al. [91] fabricated the double-clamped and cantilever resonators using a thin silicon layer structure on the 10-micrometer PI film through the manufacturing method compatible with the standard surface MEMS processing technology. The 10-micrometer-thick PI substrate strip of resonators is mounted on a printed circuit board (PCB). The reliable process of flexible thin film silicon micro resonators was successfully developed, and no structural damage was found when the flexible micro resonator was subjected to slight flexible deformations.

**Figure 7:**
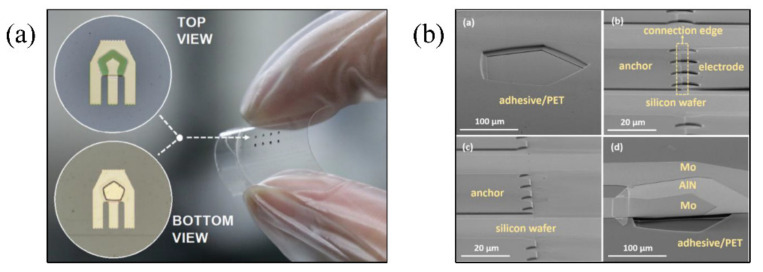
(**a**) Image of a flexible substrate carrying thin film resonators and local microscopy images of the resonator cavity [94] Reprinted/adapted with permission from Ref. [94]. 2017, AIP Publishing. (**b**) SEM image of the details of the flexible resonator [94] Reprinted/adapted with permission from Ref. [94]. 2017, AIP Publishing.

Yu et al. [96] reported the development of flexible surface-integrated FBARs fabricated on PET substrates, and proposed a novel filter structure with high performance. The flexible deformation of the surface FBARs under bending conditions was studied. The results showed that the resonant frequency of the FBARs is 1.2 GHz, and the effective electromechanical coupling coefficient is around 4.7%. The filter-like design increased the FBAR’s quality factor from 19.6 to as high as 164.2, and the devices can still function well after being subjected to hundreds of large-angle bends. Jiang et al. [94] fabricated an air-gap thin film PZT MEMS resonator with a frequency of 2.6 GHz based on a PET substrate. The fabrication process combines transfer printing and hot-embossing in order to prepare the unsupported structure. Figure 7a [94] shows the image of the thin resonator on the flexible substrate in addition to the local microscopy view of the cavity structure of the resonator. SEM images of the cavity formed on the adhesive by a hot-embossing process, the anchor structure of connection between the FBAR and the silicon substrate, the anchor structure remaining on the silicon wafer after the transfer processing, and the FBAR transferred onto the flexible substrate adhesive are all shown in Figure 7b [94]. The quality factor of the flexible resonator is 946, and the effective coupling coefficient is 5.1%. Its high performance still remains under a flexible bending deformation of 1 cm.

**Figure 8:**
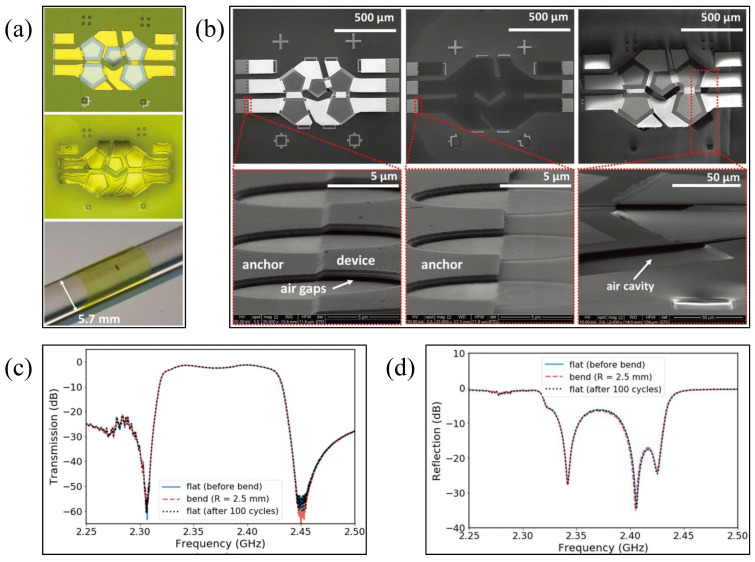
(**a**) Microscopic images of the flexible filter and the bent flexible filter [98] Reprinted/adapted with permission from Ref. [98]. 2018, John Wiley and Sons. (**b**) SEM images of the flexible filter [98] Reprinted/adapted with permission from Ref. [98]. 2018, John Wiley and Sons. (**c**) Transmission (S21) and (**d**) reflection (S11) performances of the flexible filter [98] Reprinted/adapted with permission from Ref. [98]. 2018, John Wiley and Sons.

Jiang et al. [98] presented a flexible-film RF filter based on FBAR structure, whose central frequency is 2.4 GHz and which consists of five cavity type FBARs. Each FBAR is composed of AlN PZT thin film sandwiched between two thin-layer electrodes. The microscope image of the flexible filter and its bending picture attached to the surface of the glass rod are shown in Figure 8a [98]. SEM images clearly show the key unsupported microstructures in the manufacturing process, as shown in Figure 8b [98]. The filter has a peak insertion loss of −1.14 dB, and a 3-decibel bandwidth of 107 MHz. During bending tests with a bending radius of 2.5 mm and after 100 bending cycles, the electrical properties of the filter did not degrade, and the mechanical integrity was not damaged, as shown in Figure 8c,d [98]. 

**Figure 9:**
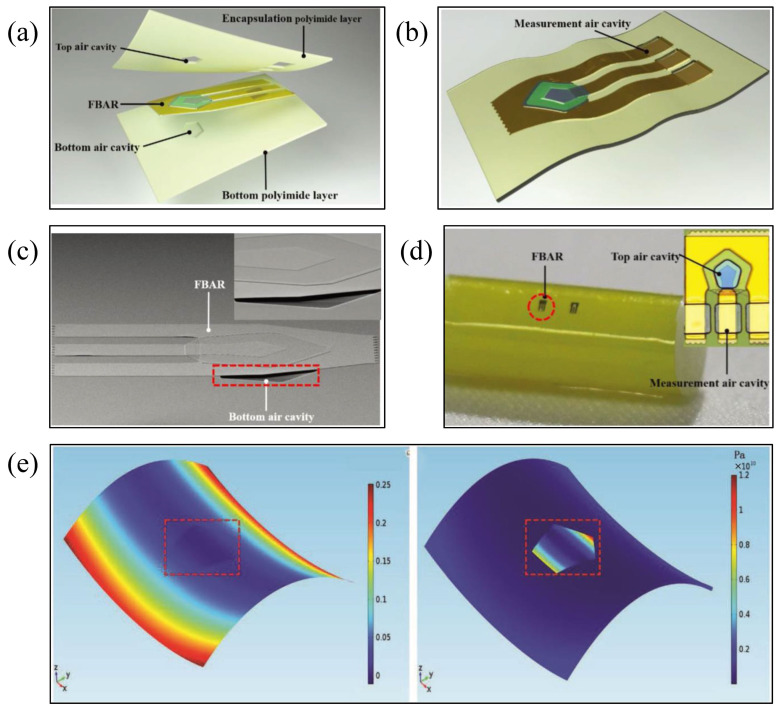
(**a**) Exploded-view image of the flexible device [100] Reprinted/adapted with permission from Ref. [100]. 2018, John Wiley and Sons. (**b**) Diagram of the FBAR embedded in double-layer PI film [100] Reprinted/adapted with permission from Ref. [100]. 2018, John Wiley and Sons. (**c**) SEM image of the FBAR with the bottom cavity structure [100] Reprinted/adapted with permission from Ref. [100]. 2018, John Wiley and Sons. (**d**) Photo of the flexible FBAR conforming to the surface of the cylinder [100] Reprinted/adapted with permission from Ref. [100]. 2018, John Wiley and Sons. (**e**) FEM analysis of the mechanical properties of the flexible FBAR under bending conditions [100] Reprinted/adapted with permission from Ref. [100]. 2018, John Wiley and Sons.

As a continuation and improvement of the above research, Zhang et al. [100] introduced a flexible FBAR that can withstand large bending deformations, and designed the FBAR structure in the neutral layer between two polyimide films in order to strictly meet the requirements of bending deformation. The exploded diagram of the flexible device, the diagram of the FBAR embedded in the double-layer PI film, the SEM image of the FBAR with the bottom cavity structure, and the photos of the flexible FBAR conforming to the surface of the cylinder with a radius of 4 mm are all shown in Figure 9a–d [100]. Cavities were created above and below the resonator in order to provide space for the free mechanical movement of the resonator structure. The flexible FBARs have a resonant frequency of 2.7 GHz, a quality factor value of 1398, and a ${k_{t}^{2}}_{\mathrm{eff}}$ of 4.39%. The minimum bending radius that the flexible resonator can withstand is about 0.5 mm. Compared with the aforementioned research, the minimum bending radius is reduced by 20 times, and the electrical performance is almost unchanged. Throughout experimental testing, the mechanical and electrical properties of the flexible resonator can still remain stable after 2000 times of bending deformation. Finite element method (FEM) analysis was utilized to evaluate the mechanical properties of the flexible device, and the strain and stress distributions of the flexible FBAR under a bending radius of 0.5 mm are shown in Figure 9e [100]. 

Han et al. proposed an MEMS beam low-pass filter based on a flexible substrate, which is implemented using a CPW and complementary split-ring resonator (CSRR) in an MEMS beam signal line. The performance changes of such filters caused by bending their flexible substrates were studied theoretically, simulatively and experimentally [102].

This section has summarized the flexible RF MEMS with different functions, mainly RF MEMS switching and flexible RF MEMS applications based on it, including phase shifters, reconfigurable antennas and phased array antennas, and resonators. This section can help to establish an overall understanding of the development status of flexible RF MEMS with different functions and structures.

## 3. Materials for Flexible RF MEMS

MEMS micro-machining technology originated in integrated circuit manufacturing, and silicon has been the main material choice. Thanks to its unique advantages, polymer substrates have become an important novel type of material used in the manufacture of flexible MEMS. Firstly, they are flexible; secondly, they are compatible with more flexible manufacturing and packaging technologies; thirdly, they are highly biocompatible; fourthly, the cost of these polymer substrates are much lower than that of monocrystalline silicon. Many flexible substrate materials have been explored for the manufacture of RF MEMS, including liquid crystal polymers (LCPs) [40,47,105,106], polyethylene terephthalate (PET) [60,94,96], polyimide (PI) [59,91,92,99,100], polydimethylsiloxane (PDMS) [58] and thinned silicon [93].

### 3.1. Liquid Crystal Polymers (LCPs)

Liquid crystal polymers are a kind of polymer material with unique and excellent structural and physical properties. They consist of interconnecting rigid and flexible monomers. When LCP flows in a molten liquid crystal, the rigid parts of the molecules are arranged next to each other along the flow direction. The LCP retains this orientation and structure even when the temperature drops below its melting point [47]. Thanks to their unique structure, LCPs have superior electromechanical properties to other flexible polymers. High-frequency test results show that LCPs have a unidirectional relative dielectric constant of 3 in the range from 0.5 to 40 GHz, and an extremely low loss factor of around 0.004 [47]. LCPs have very low moisture absorption of around 0.02% and low moisture permeability [47]. Therefore, they can be used as a base material and moisture-resistant packaging material for flexible RF MEMS switches [54,56]. 

A cantilever beam MEMS switch developed by Wang et al. [47] was integrated onto an LCP flexible substrate. Wang et al. [49] developed the air-bridge CPW switch, which can integrate 3D RF front-end reconfigurable architecture on LCPs. Han’s team [57,66,67,102] conducted a series of flexible RF MEMS studies based on LCP substrates, including double-clamped fixed MEMS switches, V-shaped thermal actuator switches, flexible low-pass filters, etc., and established corresponding theoretical models based on these devices in order to study their mechanical and electrical performances under bending states. The research shows that LCP-based switches can be used as low-cost or enhanced performance alternatives to silicon-based RF MEMS switches.

A flexible RF MEMS phase shifter proposed by Nickolas et al. [78] uses LCP material as the packaging layer. The loss measurement and mechanical measurement show that the influence of LCP packaging on the performance of the phase shifter can be ignored, but it can maintain the equipment’s flexibility and protect it against various environmental conditions. LCPs are not only used in flexible RF MEMS, but also widely used in various flexible conformal RF devices and in various flexible MEMS sensors [107,108,109,110,111,112,113,114,115].

### 3.2. Polyethylene Terephthalate (PET)

PET film is suitable to use as a flexible substrate because of its excellent impact strength, thermal stability and good bending properties [116]. Moreover, PET film has a relatively high dielectric constant and a low microwave loss tangent, which may lead to size reduction. When characterized with the microstrip T resonator method, the dielectric constant and loss angle tangent of the PET film are 3.2 and 0.01, respectively, at about 2 GHz [117].

Rivadeneyra et al. [60,118] fabricated cantilever MEMS switches on PET substrates using a screen-printing method, which demonstrated the excellent ability of PET substrates to ensure good flexibility. Molina-Lopez et al. [119] fabricated MEMS microbridges on PET substrates using a simple four-step process. Large area array fabrication of MEMS microbridges was realized using inkjet printing, and the products exhibited good flexibility and electrical characteristics.

Yu et al. [96] designed and fabricated a flexible FBAR with a frequency of 1.2 GHz by directly surface micro-machining on a PET substrate, while Jiang et al. [94] designed and fabricated a flexible FBAR with a frequency of 2.6 GHz on a PET substrate with an air cavity design by using a flexible transfer printing method. Throughout testing, these two FBARs both showed good RF and flexible performance.

### 3.3. Polyimide (PI)

Polyimide has a variety of outstanding properties, including a high glass transition temperature, excellent thermal stability, low dielectric constant, good mechanical properties, low moisture absorption, chemical stability and solvent resistance [40,120], all of which determine that it can be used in many fields, such as in flexible substrate materials [64,121,122,123], flexible device structure materials [59,124,125,126], flexible packaging materials [127,128], etc.

The flexible RF MEMS with cantilever structure proposed by Petroni et al. [92] and Pestana et al. [91] is developed based on the surface MEMS processing of flexible PI substrate. Petroni et al. [92] studied the mechanical and resonance response characteristics of cantilever resonant structures using residual stress. Pestana et al. [91] mounted the flexible resonator on a 10-micron-thick PI substrate to a PCB.

Pang’s team [98,99,100] proposed a series of flexible RF resonators based on PI substrate. Jiang et al. [98] presented a flexible RF filter with a central frequency of 2.4 GHz based on FBARs, which consists of five cavity FBARs, each comprised of an AlN PZT thin film sandwiched between two thin-layer electrodes. In the bending tests with a bending radius of 2.5 mm and after 100 bending cycles, the electrical properties of the filter did not degrade and the mechanical integrity was not damaged. In order to improve bending characteristics, Zhang et al. [100] placed the FBAR mechanical neutral plane of two polyimide thin films to fabricate a highly bendable FBAR, which demonstrated stable electrical performance under flexible deformation with a bending radius of 0.5 mm. Sun et al. [99] proposed a flexible Lamb wave resonator (LWR) based on a PI substrate that showed excellent and stable electrical properties and performed well in the bending test.

In flexible RF MEMS, PI film can also be used as the structural layer material of membrane beams. The film is commonly utilized in RF MEMS switches manufactured by printed circuit technology [59,124,125,126]. The key features of these switches are the following: (1) high dimensional tolerances and accuracy can be achieved using traditional printed circuit machining processes; (2) mass fabrication of such switches can be completed using low-cost PCB processing technology; (3) simple integration with RF components using proven integration and packaging technologies; (4) large numbers of such devices can be easily integrated into large areas.

In addition to the above three kinds of flexible substrate materials, there are other flexible materials applied in flexible RF MEMS, such as PDMS, SU-8, thin silicon, etc., although few relevant application achievements can be retrieved. For example, Shah et al. [58] introduced an elastomer pneumatic switch for RF micro-devices based on PDMS. Compared with being a flexible substrate material, the usual function of PDMS is to act as a stamp in the transfer printing process [97,98,99,100]. Chao et al. [65] developed an SU-8 micro-machining process for RF MEMS passive preparation. The beam of the switch is made of an SU-8/Cu/SU-8 sandwich structure, in order to effectively relieve the effects of residual stress on device properties. The flexible FBAR based on ultra-thin silicon wafer proposed by Kang et al. [93] is fabricated using a standard MEMS process, with high machining accuracy and good device performance. However, compared with other flexible substrates, the thinned silicon wafer can only withstand small bending conditions as a result of its poor ductility.

This section has summarized the three flexible materials most commonly used by flexible RF MEMS, LCP, PET and PI. Among them, LCP has been widely used because of its excellent mechanical and electrical characteristics.

## 4. Fabrication Processing of Flexible RF MEMS

The fabrication techniques of flexible RF MEMS devices can be divided into the following three strategies: (1) surface MEMS processing techniques directly fabricated on various flexible substrates [47,48,57,66,67,69,70,76,77,78,91,92,93,96,102]; (2) transfer processing techniques of transferring fabricated RF MEMS from rigid substrates to flexible substrates [60,62,63,68,94,97,98,99,100,101]; and (3) direct printing inkjet printing processing techniques of functional electronic materials [53,59,60,61,64,71,72,73].

### 4.1. Surface MEMS Processing

According to the types of substrates, surface processing technologies based on flexible substrates can be divided into the following two categories: flexible polymer substrate surface processing technology, and thinned silicon substrate surface processing technology.

**Figure 10:**
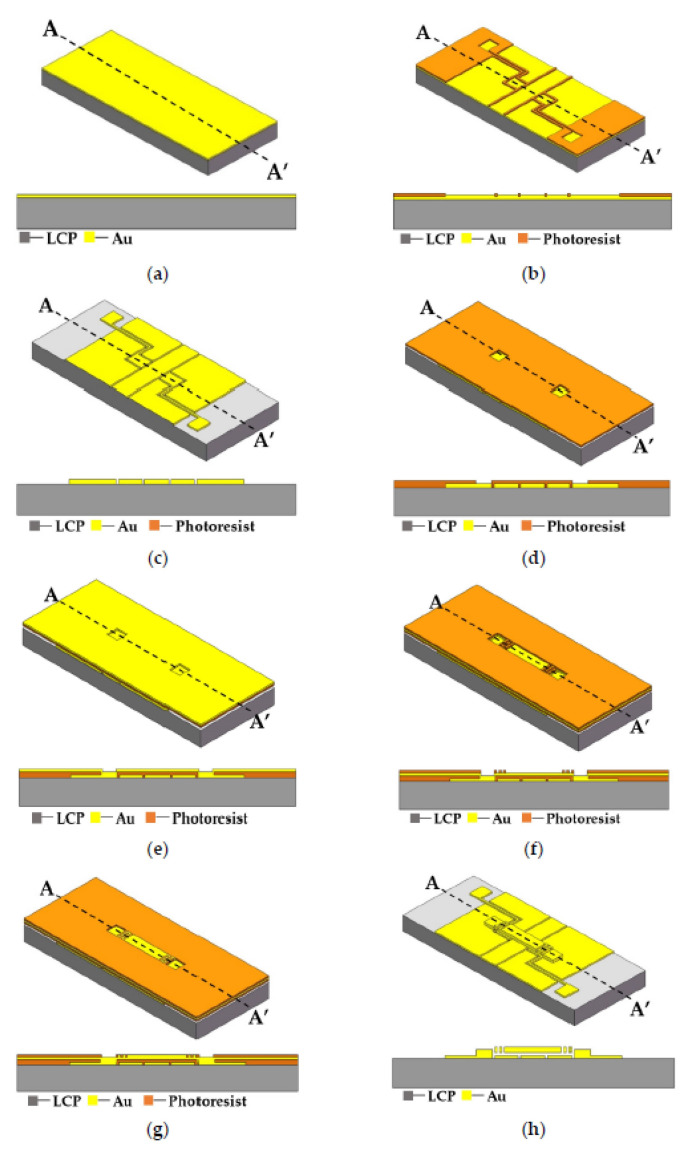
Surface processing technology based on flexible LCP substrate [67]. (**a**) Sputter the CPW seed layer; (**b**) Pattern the seed layer and electroplate; (**c**) Remove the redundant seed layer; (**d**) Pattern the anchor photoresist; (**e**) Sputter the beam seed layer; (**f**) Pattern the beam photoresist; (**g**) Electroplate the beam; (**h**) Release the structure.

Surface MEMS processing involves directly growing devices onto flexible polymer substrates, and the method is shown in Figure 10 [67]. The obvious advantage of this processing technology is that it is compatible with the existing surface micro-machining process, and the fabricated electronics have good flexible bending performance. The disadvantage of this technology is that due to the introduction of flexible polymer substrate materials, the existing process may need to be modified. At the same time, the yield of the fabricated flexible RF MEMS is not high, and the performance of some devices will be degraded.

For thinned silicon substrate surface processing technology, its advantage is that the processing accuracy, device performance, process compatibility, yield and other aspects can be fully guaranteed. The flexible resonator fabricated based on 50-micrometer thinned silicon using this technology is shown in Figure 6a–c [93]. The disadvantage of this technology is that the thinned silicon wafer can only withstand small bending deformations as a result of its poor ductility.

### 4.2. Transfer Processing

According to different transfer methods, there are two specific implementation strategies for transfer technology: rigid chip transfer technology and flexible transfer technology using transfer stamping.

**Figure 11:**
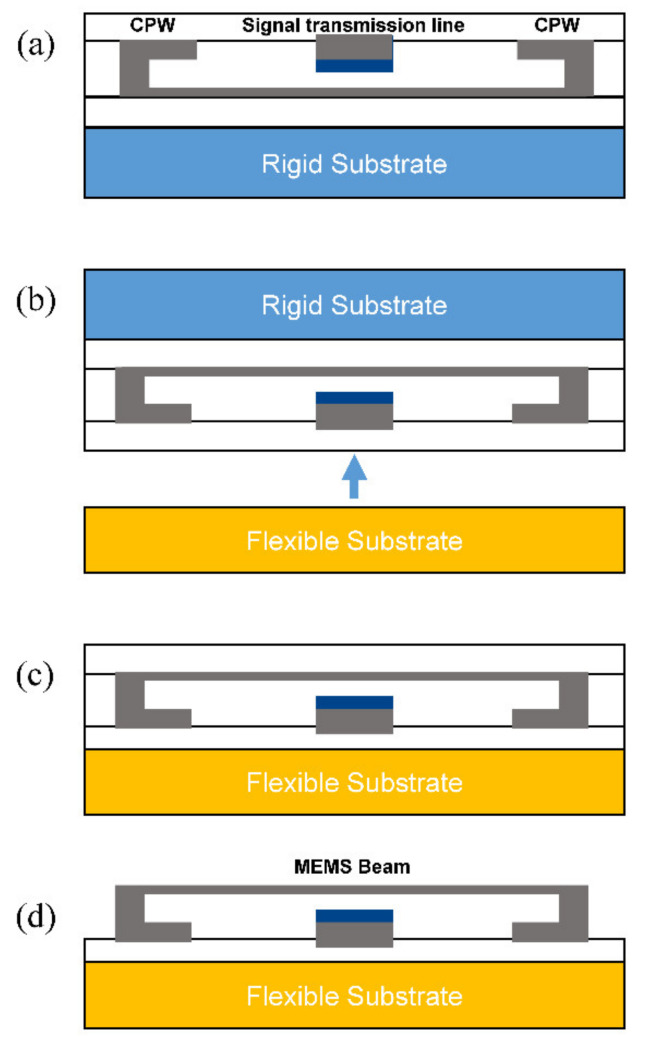
Wafer transfer technology (WTT) process flow for MEMS switch fabrication. (**a**) Reversed process sequence; (**b**) Transfer process; (**c**) Remove the silicon substrate; (**d**) Release the structure.

Zhang et al. [63] presented a flexible MEMS switch based on wafer transfer technology, and its process flow is shown in Figure 11 [63]. The RF MEMS switch structure is first prepared on a rigid silicon wafer by reverse placement through the standard MEMS process, and then the switch is transferred to the flexible substrate through the bond sum, silicon wafer thinning and wet etching processes. Finally, the switch is formed through the release process. The advantages of this processing technology are compatible with the standard MEMS process, with high machining accuracy, and the prepared flexible RF MEMS switch retains the good flexibility of the substrate. This technology has the disadvantages of being a complex process, with low yields and high costs.

As a novel flexible electronics manufacturing technology, transfer printing can use soft stamping to assemble multi-scale and category devices, which is a promising strategy [129,130,131,132,133,134]. Recent research achievements have proved that applying this fabrication technology to the fabrication of flexible RF MEMS can be effective.

**Figure 12:**
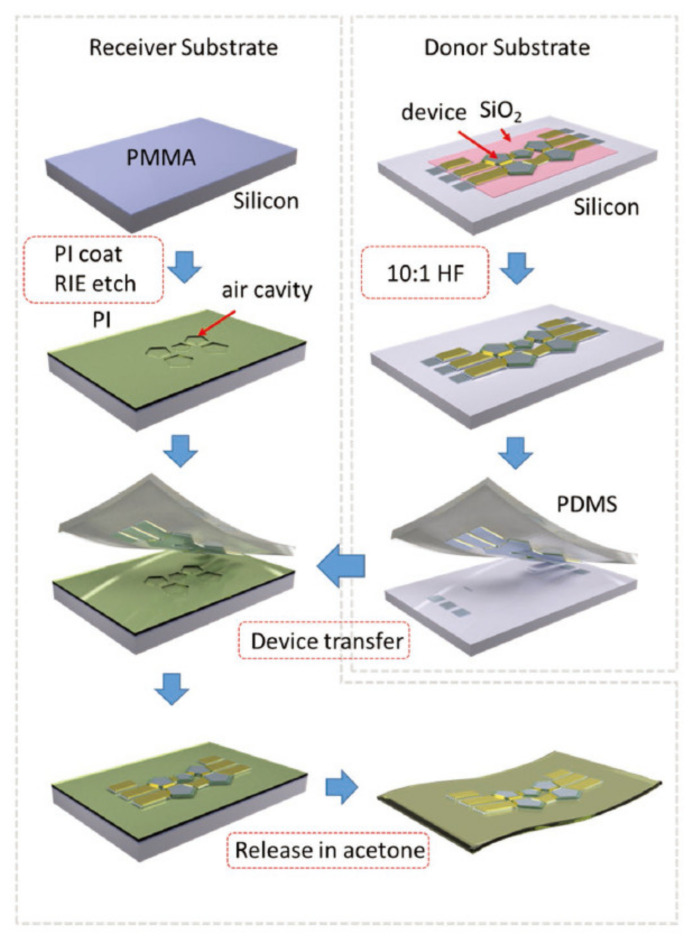
Transfer printing technology process flow for RF MEMS fabrication [98] Reprinted/adapted with permission from Ref. [98]. 2018, John Wiley and Sons.

Pang’s team [98,99,100] proposed a series of flexible RF resonators based on PI substrate using the transfer printing technology. The transfer printing technology process flow for flexible RF MEMS fabrication is shown in Figure 12 [98]. Combined with appropriate device structure design, strain isolated cavity design and mechanical neutral layer design [98,100], flexible RF MEMS based on transfer printing technology have very excellent flexible deformation characteristics and can meet conformal scenes with large curvatures. However, the disadvantages of this technology are that it is not compatible with the existing process, the process maturity needs to be improved, the yield is low, and it is difficult to mass produce.

### 4.3. Inkjet Printing

Direct inkjet printing of functional electronic materials may provide a novel route to manufacturing integrated circuits, with its advantages of low-cost equipment, flexible substrate, low-cost materials, degradability and biocompatibility [135,136,137,138]. Compared with the technologies discussed above, inkjet printing technology is an efficient and mask-free micro-processing technology based on additive manufacturing. It is especially suitable for micro/nano fabrication experiments with new structures and functional design verification. Using this technology to fabricate flexible RF MEMS can improve the research efficiency of the performance and innovation of fabrication technologies. Takao’s team [71,72,73] proposed a plastic MEMS switch based on inkjet printing and subsequently, a flexible wireless power transmission system based on this switch. Not only does a single flexible MEMS switch show the stability of driving performance and electrical performance [71], but also flexible wireless power transmission system combined with organic transistors realizes the application of light, flexible, large-area and high-power transmission [72,73]. Inkjet printing technology has some disadvantages in the fabrication of flexible RF MEMS. Printing technology is not compatible with standard surface MEMS processing. Moreover, it is difficult to control print uniformity, especially at the micro/nano scale, and the resolution is a principal limiting factor. The printing speed is slow, and batch production is difficult. Printing equipment has high requirements for printable materials.

This section has summarized three commonly used fabrication strategies of flexible RF MEMS. (1) Surface MEMS processing technology directly prepared on a variety of flexible substrates has the advantages of compatibility with existing processes; (2) the transfer processing technology of RF MEMS fabricated from rigid substrates to flexible substrates has the advantages of flexible fabrication processing and high flexibility electronics; (3) the direct inkjet printing processing technology of functional electronic materials is very suitable for the experimental verification of new structures without masks.

## 5. Novel 3D RF Mechanical Self-Assembly and Mechanically Triggered Switches

Most of the flexible RF MEMS fabricated by the strategies discussed above can only bear flexible bending deformation, but not tensile deformation. In addition, the flexible deformation is only borne by the flexible substrate, and it is not expected that the structure of RF MEMS devices will change significantly, so as to ensure the stability and reliability of RF MEMS devices. The recently developed flexible 3D electronic device assembly technology based on mechanical pre-stretching guidance uses mature semiconductor technology to integrate with pre-stretched flexible substrates to form 2D precursor structures. By releasing the pre-stretching strain, the device structure produces compression buckling, and realizes the controlled conversion from 2D structures to 3D structures. Compared with other 3D device manufacturing technologies, the assembly and control functions of flexible stretching are used to improve the stretchability of flexible electronics, expand the application scope of flexible 3D electronics, and provide innovative preparation strategies for flexible 3D electronics [139,140,141,142,143,144,145,146,147,148,149,150,151,152,153].

**Figure 13:**
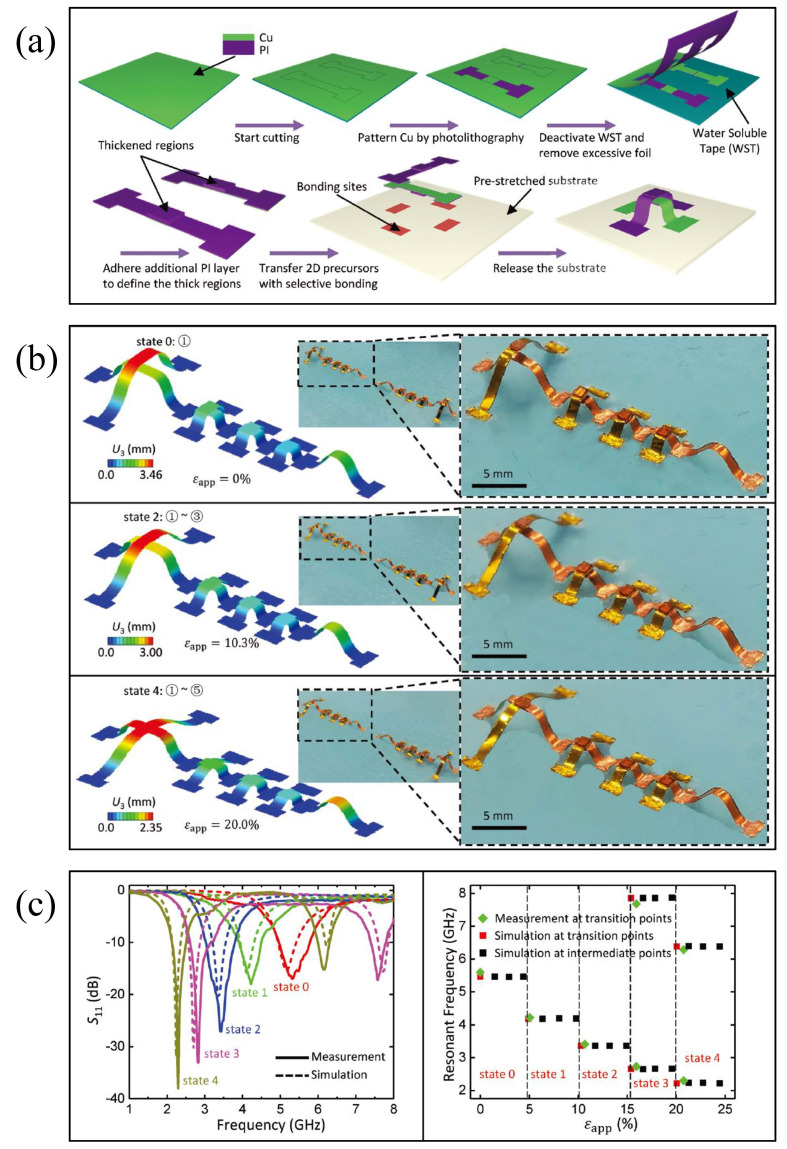
(**a**) Image of the preparation process for the mechanically guided 3D assembly switch [143] Reprinted/adapted with permission from Ref. [143]. 2019, John Wiley and Sons. (**b**) FEM and optical images of the antenna under different tensile states [143] Reprinted/adapted with permission from Ref. [143]. 2019, John Wiley and Sons. (**c**) Measured and simulated characteristics of the antenna at different states [143] Reprinted/adapted with permission from Ref. [143]. 2019, John Wiley and Sons.

Liu et al. [143] presented stretchable and reconfigurable 3D RF antenna designs based on mechanically triggered switches that utilize controlled compressive buckling to form the devices from patterned 2D structures integrated with an elastic substrate. The mechanically guided assembly technique is used to design tandem integrated switches, in order to form scalable and reconfigurable antennas. The mechanically triggered switch consists of two arched belt structures of different heights that switch to the open state when the two arched structures touch each other. The switch state can be actively controlled by the tensile strain applied to the substrate. Through a cascaded switching strategy, the reconfigurable antenna can adjust its frequency in the range of 2.3 to 7.7 GHz, as shown in Figure 13 [143].

This flexible reconfigurable 3D RF antenna based on mechanical self-assembly and mechanical triggering will not degrade its RF characteristics under tensile strain, but takes it as a trigger condition to actively manipulate the RF characteristics. At present, the reported reconfigurable 3D devices based on this mechanical assembly and regulation are on a millimeter scale; there is no relevant research on micro/nano scale RF devices. The means to realize the application of this strategy in the fabrication of micro- and nanoscale devices may be a research and discussion direction.

## 6. Conclusions and Perspectives

This paper provided an overview of recent achievements in the structural design, fabrication process and performance optimization of flexible RF MEMS. From the perspective of device function, flexible RF MEMS can be sorted into the following types: RF switches, phase shifters, reconfigurable and phased array antennas, resonators, etc. Flexible polymer films, such as LCP, PI, PET, etc., are widely used as flexible RF MEMS substrates, and a small part of the substrates are composed of thinned silicon wafers. The fabrication techniques of flexible RF MEMS include the following: surface MEMS processing techniques, transfer processing techniques and inkjet printing processing techniques. A systematic summary of the functions, materials and fabrication processes of flexible RF MEMS can help researchers select appropriate structural designs, materials and compatible fabrication processes according to the required application scenarios and functions of flexible RF MEMS. In addition, 3D RF devices based on mechanical self-assembly and mechanically triggered switches are discussed, which provide novel strategies for flexible, reconfigurable 3D RF device fabrication processes. This review serves the purpose of providing a reference source for innovative research in the field of flexible RF MEMS.

In terms of future research directions of flexible RF MEMS, we consider three directions worthy of breakthrough: firstly, to carry out material innovation and develop new functional materials with flexible and RF properties that are more suitable for flexible RF MEMS; secondly, to innovate device structural design in order to improve the competitiveness of flexible RF MEMS and rigid RF MEMS in performance, and to deal with the interaction between flexible substrate deformation and device performance, which should be a focus of future research in this field; thirdly, to improve the reliability of flexible RF MEMS devices and systems, which is a problem that must be solved in order to achieve large-scale applications in this field.

## Figures and Tables

**Table 1:** Comparison of RF MEMS/Flexible MEMS reviews.

Review Theme	Rigid or Flexible	Application	Substrate Materials	Structural Materials	Year	Ref.
RF MEMS	Rigid	MEMS Switches in Different Frequency Bands	Si/SiO_2_/GaAs	Metal	2018	[14]
RF MEMS	Rigid	Piezoelectric MEMS Resonators	Si	Piezo	2021	[15]
RF MEMS	Rigid	Carbon-Based MEMS/NEMS	Si	Metal/Piezo/Carbon-Based materials	2011	[16]
RF MEMS	Rigid	MEMS/NEMS Electrostatic Actuation Stability	Si	Si/Metal	2014	[17]
RF MEMS	Rigid	RF MEMS Switch with Low Electrostatic Drive Voltage	Si	Metal and Carbon Alloy	2013	[18]
RF MEMS	Rigid	COMS Compatible Passive RF Devices	Si/SOI (Silicon-on-Insulator)	Si	2016	[19]
RF MEMS	Rigid	Integrated Magnetic MEMS Relays	Si	Magnetic Metal	2014	[20]
RF MEMS	Rigid	Electrostatically driven NEMS Switches	Si/SOI	Si/SiN_x_/SiC/Metal	2018	[21]
RF MEMS	Rigid	Mechanical Effects on RF MEMS Switches	Si	Si/Metal	2020	[22]
RF MEMS	Rigid	Micro-machining of Silicon-based MEMS Resonators	Si/SOI/SiC	Si/Metal	2021	[23]
RF MEMS	Rigid	Electrothermal MEMS Actuators	Si	Metal/Si/CNT (carbon nanotube)	2019	[24]
RF MEMS	Rigid	RF MEMS Performance Drift Reliability Problems	Si/Glass	Si/Metal	2016	[25]
RF MEMS	Rigid	RF MEMS Switch High-Performance Material and Manufacturing Method	Si/Glass/GaAs/SOI	Metal	2021	[26]
RF MEMS	Rigid	RF MEMS Switch Principles, Characteristics and Requirements	Si/Glass/GaAs	Si/Metal	2014	[27]
RF MEMS	Rigid	RF MEMS Inductor Performance	Si/Glass	Si/Metal	2017	[28]
RF MEMS	Rigid	Reconfigurable Metamaterials and Antennas Based on MEMS	Si/Quartz	Si/Metal	2014	[29]
RF MEMS	Rigid	RF MEMS Switch with Different Driving Principle	Si/Glass/SOI	Si/Metal/PZT	2020	[30]
RF MEMS	Rigid	Application of Gold Materials in RF MEMS Membranes	LaAlO_3_	Au	2016	[31]
RF MEMS	Rigid	Structure, actuation methods, contact types of RF MEMS switches	Si	Metal	2021	[32]
Flexible MEMS	Flexible	Flexible MEMS Supercapacitors	PET/PI/PDMS/Si	PVA-H_3_PO_4_/Ionogel/Metal/ CNT	2016	[33]
Flexible MEMS	Flexible	MEMS Direct Write Fabrication Technology	Si/Polymer	Polymer/Metal Nano Composites	2017	[34]
Flexible MEMS	Flexible	MEMS-Based Flexible Skin	Polymer/Si/	Metal/Polymer/Si	2013	[35]
Flexible MEMS	Flexible	MEMS Electromagnetic Actuators for Biomedical Applications	PMMA/PDMS/PI/ Parylene	PZT/Metal/Magnetic Metal	2020	[36]
Flexible MEMS	Flexible	Flexible MEMS Tactile Sensors for Robots	Si/PVDF/Kapton	Metal/PZT/SiN_x_	2017	[37]
Flexible MEMS	Flexible	Flexible Capacitive MEMS Pressure Sensors	PET/PI/Paper/Si	PDMS/ITO/Metal/ Graphene	2021	[38]
Flexible MEMS	Flexible	MEMS of Polymers as Structural Materials	PDMS/PI/PET	PDMS/PVDF/LCP/ SU-8/Epoxy Composites	2018	[39]
Flexible MEMS	Flexible	Polymer MEMS Processing Technology	PDMS/PI/PET/Si	PDMS/ SU-8/PI/ Parylene C	2016	[40]
Flexible MEMS	Flexible	Rigid MEMS Flexible Trends	Polymer/Si	PZT/Graphene/Metal/Si	2020	[41]
Flexible MEMS	Flexible	Mechanically Driven Reconfigurable Metasurfaces	PDMS/SU-8/ PMMA/Si	Metal/SU-8/PDMS	2021	[42]
Flexible MEMS	Flexible	Microthermofluidic Systems for Biochemical	PDMS/PI/PET/PP	PDMS/PI/PET/PP/PZT	2022	[43]
Flexible MEMS	Flexible	MEMS-Based Cantilever Biosensors	PDMS	PZT/Metal/ SiO_2_/PEDOT:PSS/PDMS	2019	[44]
Flexible MEMS	Flexible	Flexible MEMS Sensors and Devices	PDMS/PI/PET/ Kapton	Metal/PZT/Si	2019	[45]

**Table 2:** Summary of flexible RF MEMS switches.

Substrate Materials	Substrate Materials	Structural Features	Fabrication Processing	Year	Ref.
LCP	Al	Cantilever Beam	Surface MEMS Processing	2003	[47]
PET	Si	Double-Clamped	Surface MEMS Processing	2008, 2007	[48]
LCP	Au	Double-Clamped	Surface MEMS Processing	2004	[49]
LCP	Cu, Au	Double-Clamped, LCP Package	Surface MEMS Processing	2007, 2005, 2008	[54,55,56]
LCP	Cu, Au	Double-Clamped	Surface MEMS Processing	2015, 2016, 2020	[57,66]
PDMS	Au	Pneumatic	Surface MEMS Processing	2012	[58]
PI	Ag	Cantilever Beam	Printed	2015	[60]
FR-4	Au	Double-Clamped	Transfer Processing	2006	[63]
Kapton	Ag/PEDOT:PSS	Double-Clamped	Printed	2018	[64]

## Data Availability

Not applicable.

## References

[1] Osseiran A., Boccardi F., Braun V., Kusume K., Marsch P., Maternia M., Queseth O., Schellmann M., Schotten H., Taoka H. (2014). Scenarios for 5G Mobile and Wireless Communications: The Vision of the METIS Project. IEEE Commun. Mag..

[2] Hwang I.J., Oh J.I., Jo H.W., Kim K.S., Yu J.W., Lee D.J. (2022). 28 GHz and 38 GHz Dual-Band Vertically Stacked Dipole Antennas on Flexible Liquid Crystal Polymer Substrates for Millimeter-Wave 5G Cellular Handsets. IEEE Trans. Antennas Propag..

[3] Hong W. (2017). Solving the 5G Mobile Antenna Puzzle: Assessing Future Directions for the 5G Mobile Antenna Paradigm Shift. IEEE Microw. Mag..

[4] Gowrish B., John D., Settu D., Basu A., Koul S.K. Novel mechanical reconfigurable PCB antenna for 2.4 GHz wireless consumer product: Minimizing time to market. Proceedings of the 2014 3rd Asia-Pacific Conference on Antennas and Propagation.

[5] Lee G.H., Moon H., Kim H., Lee G.H., Kwon W., Yoo S., Myung D., Yun S.H., Bao Z., Hahn S.K. (2020). Multifunctional materials for implantable and wearable photonic healthcare devices. Nat. Rev. Mater..

[6] Zhang H., Lan Y., Qiu S., Min S., Jang H., Park J., Gong S., Ma Z. (2020). Flexible and Stretchable Microwave Electronics: Past, Present, and Future Perspective. Adv. Mater. Technol..

[7] Dudala S., Dubey S.K., Goel S. (2019). Fully Integrated, Automated, and Smartphone Enabled Point-of-Source Portable Platform with Microfluidic Device for Nitrite Detection. IEEE Trans. Biomed. Circuits Syst..

[8] Xiong W.N., Zhu C., Guo D.L., Hou C., Yang Z.X., Xu Z.Y., Qiu L., Yang H., Li K., Huang Y.A. (2021). Bio-inspired, intelligent flexible sensing skin for multifunctional flying perception. Nano Energy.

[9] Liu J., Jiang S., Xiong W., Zhu C., Li K., Huang Y. (2021). Self-Healing Kirigami Assembly Strategy for Conformal Electronics. Adv. Funct. Mater..

[10] Zhang S.H., Zhu J., Zhang Y.Y., Chen Z.S., Song C.Y., Li J.Q., Yi N., Qiu D.H., Guo K., Zhang C. (2022). Standalone stretchable RF systems based on asymmetric 3D microstrip antennas with on-body wireless communication and energy harvesting. Nano Energy.

[11] Chung H.U., Kim B.H., Lee J.Y., Lee J., Xie Z.Q., Ibler E.M., Lee K., Banks A., Jeong J.Y., Kim J. (2019). Binodal, wireless epidermal electronic systems with in-sensor analytics for neonatal intensive care. Science.

[12] Huang Y., Wu H., Zhu C., Xiong W., Chen F., Xiao L., Liu J., Wang K., Li H., Ye D. (2021). Programmable robotized ‘transfer-and-jet’ printing for large, 3D curved electronics on complex surfaces. Int. J. Extrem. Manuf..

[13] Rebeiz G.M., Muldavin J.B. (2001). Rf Mems Switches and Switch Circuits. IEEE Microw. Mag..

[14] Tian W.C., Li P., Yuan L.X. (2018). Research and Analysis of MEMS Switches in Different Frequency Bands. Micromachines.

[15] Pillai G., Li S.S. (2021). Piezoelectric MEMS Resonators: A Review. IEEE Sens. J..

[16] Liao M.Y., Koide Y. (2011). Carbon-Based Materials: Growth, Properties, MEMS/NEMS Technologies, and MEM/NEM Switches. Crit. Rev. Solid State Mater. Sci..

[17] Zhang W.M., Yan H., Peng Z.K., Meng G. (2014). Electrostatic pull-in instability in MEMS/NEMS: A review. Sens. Actuator A Phys..

[18] Mafinejad Y., Kouzani A., Mafinezhad K. (2013). Review of low actuation voltage RF MEMS electrostatic switches based on metallic and carbon alloys. Inf. Midem J. Microelectron. Electron. Compon. Mater..

[19] Li X.X., Ni Z., Gu L., Wu Z.Z., Yang C. (2016). Micromachined high-performance RF passives in CMOS substrate. J. Micromech. Microeng..

[20] Schiavone G., Desmulliez M.P.Y., Walton A.J. (2014). Integrated Magnetic MEMS Relays: Status of the Technology. Micromachines.

[21] Jasulaneca L., Kosmaca J., Meija R., Andzane J., Erts D. (2018). Review: Electrostatically actuated nanobeam-based nanoelectromechanical switches-materials solutions and operational conditions. Beilstein J. Nanotechnol..

[22] Lysenko I.E., Tkachenko A.V., Ezhova O.A., Konoplev B.G., Ryndin E.A., Sherova E.V. (2020). The Mechanical Effects Influencing on the Design of RF MEMS Switches. Electronics.

[23] Verma G., Mondal K., Gupta A. (2021). Si-based MEMS resonant sensor: A review from microfabrication perspective. Microelectron. J..

[24] Potekhina A., Wang C.H. (2019). Review of Electrothermal Actuators and Applications. Actuators.

[25] Zhou W., He J.B., He X.P., Yu H.J., Peng B. (2016). Dielectric charging induced drift in micro device reliability—A review. Microelectron. Reliab..

[26] Kurmendra, Kumar R. (2021). Materials Selection Approaches and Fabrication Methods in RF MEMS Switches. J. Electron. Mater..

[27] Jaafar H., Beh K.S., Yunus N.A.M., Hasan W.Z.W., Shafie S., Sidek O. (2014). A comprehensive study on RF MEMS switch. Microsyst. Technol..

[28] Hikmat O.F., Ali M.S.M. (2017). RF MEMS Inductors and Their Applications-A Review. J. Microelectromech. Syst..

[29] Debogovic T., Perruisseau-Carrier J. (2014). MEMS-Reconfigurable Metamaterials and Antenna Applications. Int. J. Antennas Propag..

[30] Cao T.T., Hu T.J., Zhao Y.L. (2020). Research Status and Development Trend of MEMS Switches: A Review. Micromachines.

[31] Noel J.G. (2016). Review of the properties of gold material for MEMS membrane applications. IET Circuits Devices Syst..

[32] Kurmendra, Kumar R. (2021). A review on RF micro-electro-mechanical-systems (MEMS) switch for radio frequency applications. Microsyst. Technol..

[33] Patil S., Lee D.W. (2019). Status review on the MEMS-based flexible supercapacitors. J. Micromech. Microeng..

[34] Teh K.S. (2017). Additive direct-write microfabrication for MEMS: A review. Front. Mech. Eng..

[35] Xu Y. (2013). Post-CMOS and Post-MEMS Compatible Flexible Skin Technologies: A Review. IEEE Sens. J..

[36] Yunas J., Mulyanti B., Hamidah I., Mohd Said M., Pawinanto R.E., Ali W., Subandi A., Hamzah A.A., Latif R., Yeop Majlis B. (2020). Polymer-Based MEMS Electromagnetic Actuator for Biomedical Application: A Review. Polymers.

[37] Abels C., Mastronardi V.M., Guido F., Dattoma T., Qualtieri A., Megill W.M., De Vittorio M., Rizzi F. (2017). Nitride-Based Materials for Flexible MEMS Tactile and Flow Sensors in Robotics. Sensors.

[38] Mishra R.B., El-Atab N., Hussain A.M., Hussain M.M. (2021). Recent Progress on Flexible Capacitive Pressure Sensors: From Design and Materials to Applications. Adv. Mater. Technol..

[39] Li Y.J. (2018). Challenges and Issues of Using Polymers as Structural Materials in MEMS: A Review. J. Microelectromech. Syst..

[40] Kim B.J., Meng E. (2016). Review of polymer MEMS micromachining. J. Micromech. Microeng..

[41] Zhu J.X., Liu X.M., Shi Q.F., He T.Y.Y., Sun Z.D., Guo X.G., Liu W.X., Bin Sulaiman O., Dong B.W., Lee C. (2020). Development Trends and Perspectives of Future Sensors and MEMS/NEMS. Micromachines.

[42] Zang G.X., Liu Z.J., Deng W.J., Zhu W.M. (2021). Reconfigurable metasurfaces with mechanical actuations: Towards flexible and tunable photonic devices. J. Opt..

[43] Kulkarni M.B., Goel S. (2022). Recent advancements in integrated microthermofluidic systems for biochemical and biomedical applications—A review. Sens. Actuator A Phys..

[44] Kurmendra, Kumar R. (2019). MEMS based cantilever biosensors for cancer detection using potential bio-markers present in VOCs: A survey. Microsyst. Technol..

[45] Yang X., Zhang M. (2021). Review of flexible microelectromechanical system sensors and devices. Nanotechnol. Precis. Eng..

[46] Bernhard J.T., Chen N., Clark R., Feng M., Liu C., Mayes P., Michielssen E., Wang R., Chorosinski L.G. Electronically reconfigurable and mechanically conformal apertures using low-voltage MEMS and flexible membranes for space-based radar applications. Proceedings of the Smart Structures and Materials 2001 Conference.

[47] Wang X.F., Engel J., Liu C. (2003). Liquid crystal polymer (LCP) for MEMS: Processes and applications. J. Micromech. Microeng..

[48] Patil S.B., Chu V., Conde J.P. (2008). Performance of thin film silicon MEMS on flexible plastic substrates. Sens. Actuators A Phys..

[49] Wang G., Thompson D., Tentzeris E.M., Papapolymerou J. (2004). Low Cost RF MEMS Switches Using LCP Substrate.

[50] Oberhammer J., Stemme G. (2004). Low-voltage high-isolation DC-to-RF MEMS switch based on an S-shaped film actuator. IEEE Trans. Electron Devices.

[51] Ramadoss R., Lee S., Lee Y.C., Bright V.M., Gupta K.C. (2003). Fabrication, assembly, and testing of RF MEMS capacitive switches using flexible printed circuit technology. IEEE Trans. Adv. Packag..

[52] Ramadoss R., Lee S., Lee Y.C., Bright V.M., Gupta K.C. (2007). MEMS capacitive series switch fabricated using PCB technology. Int. J. Rf Microw. Comput.-Aided Eng..

[53] Yokota T., Nakano S., Sekitani T., Someya T. (2008). Plastic complementary microelectromechanical switches. Appl. Phys. Lett..

[54] Morton M.A., Kingsley N., Papapolymerou J. Low Cost Method for Localized Packaging of Temperature Sensitive Capacitive RF MEMS Switches in Liquid Crystal Polymer. Proceedings of the 2007 IEEE/MTT-S International Microwave Symposium.

[55] Thompson D., Kingsley N., Wang G.A., Papapolymerou J., Tentzeris M.M. RF characteristics of thin film liquid crystal polymer (LCP) packages for RF MEMS and MMIC integration. Proceedings of the IEEE Mtt-S International Microwave Symposium.

[56] Kingsley N., Bhattacharya S.K., Papapolymerou J. (2008). Moisture Lifetime Testing of RF MEMS Switches Packaged in Liquid Crystal Polymer. IEEE Trans. Compon. Packag. Technol..

[57] Gao X., Lei H., Meng N., Huang Q. The Study of a RF MEMS Switch Based on LCP Substrate. Proceedings of the IEEE Mtt-S International Microwave Symposium.

[58] Shah C.M., Sriram S., Bhaskaran M., Nasabi M., Thach Giang N., Rowe W.S.T., Mitchell A. (2012). Elastomer-Based Pneumatic Switch for Radio Frequency Microdevices. J. MicroelectroMech. Syst..

[59] Ramadoss R., Lee S., Lee Y.C., Bright V.M., Gupta K.C. (2006). RF-MEMS capacitive switches fabricated using printed circuit processing techniques. J. MicroelectroMech. Syst..

[60] Rivadeneyra A., Fernandez-Salmeron J., Agudo-Acemel M., Lopez-Villanueva J.A., Capitan-Vallvey L.F., Palma A.J. (2015). Improved manufacturing process for printed cantilevers by using water removable sacrificial substrate. Sens. Actuator A Phys..

[61] Lee S., Ramadoss R., Buck M., Bright V.M., Gupta K.C., Lee Y.C. (2004). Reliability testing of flexible printed circuit-based RF MEMS capacitive switches. Microelectron. Reliab..

[62] Zhang Q.X., Yu A.B., Guo L.H., Kumar R., Teoh K.W., Liu A.Q., Lo G.Q., Kwong D. (2006). RF MEMS switch integrated on printed circuit board with metallic membrane first sequence and transferring. IEEE Electron Device Lett..

[63] Zhang Q.X., Yu A.B., Guo L.H., Kumar R., Teoh K.W., Liu A.Q., Lo G.Q., Kwong D.L. (2006). Development of RF MEMS switch on flexible organic substrate with wafer transfer technology (WTT). Proceedings of the 56th Electronic Components & Technology Conference.

[64] Monne M.A., Lan X., Zhang C.B., Chen M.Y.H. (2018). Inkjet-Printed Flexible MEMS Switches for Phased-Array Antennas. Int. J. Antennas Propag..

[65] Chao T.-Y., Hsu M.C., Lin C.D., Cheng Y.T. (2011). SU-8 serial MEMS switch for flexible RF applications. J. Micromech. Microeng..

[66] Han L., Gao X.F. (2016). Modeling of Bending Characteristics on Micromachined RF MEMS Switch Based on LCP Substrate. IEEE Trans. Electron Devices.

[67] Han L., Chen L.J., Qin R.J., Wang K., Zhang Z.Q., Nie M., Huang X.D. (2020). Multi-Physical Models of Bending Characteristics on the Double-Clamped Beam Switch for Flexible Electronic Devices Application. Sensors.

[68] Fujishiro A., Takahashi S., Sawada K., Ishida M., Kawano T. (2014). Flexible Neural Electrode Arrays with Switch-Matrix Based on a Planar Silicon Process. IEEE Electron Device Lett..

[69] Han L., Yu Y., Qin R.J., Zhang Z.Q., Su S. (2019). Static Modeling of Bending Characteristics on V-Shaped Beam Actuator Based on Flexible Substrate. IEEE Trans. Electron Devices.

[70] Millet O. A new RF MEMS technology enabling tunability for RF front-end RF ohmic relay based on a flexible anchorless membrane. Proceedings of the 42nd European Microwave Conference.

[71] Nakano S., Sekitani T., Yokota T., Someya T. (2008). Low operation voltage of inkjet-printed plastic sheet-type micromechanical switches. Appl. Phys. Lett..

[72] Sekitani T., Takamiya M., Noguchi Y., Nakano S., Kato Y., Sakurai T., Someya T. (2007). A large-area wireless power-transmission sheet using printed organic transistors and plastic MEMS switches. Nat. Mater..

[73] Sekitani T., Noguchi Y., Nakano S., Zaitsu K., Kato Y., Takamiya M., Sakurai T., Someya T. (2007). Communication sheets using printed organic nonvolatile memories. Proceedings of the IEEE International Electron Devices Meeting.

[74] Liu J.S., Shu Z., Wang C., Shan H., Yin P.H., Zhang F.T., Zhang Y., Xiong Z., Du L.Q., Tang B. (2018). Fabrication of a flexible polyimide-based electrostatically actuated MEMS relay. J. Micromech. Microeng..

[75] Touati S., Lorphelin N., Kanciurzewski A., Robin R., Rollier A.S., Millet O., Segueni K. Low Voltage Totally Free Flexible RF MEMS Switch with Anti-Stiction System. Proceedings of the 2008 Symposium on Design, Test, Integration and Packaging of MEMS/MOEMS.

[76] Chung D.J., Polcawich R.G., Pulskamp J.S., Papapolymerou J. (2012). Reduced-Size Low-Voltage RF MEMS X-Band Phase Shifter Integrated on Multilayer Organic Package. IEEE Trans. Compon. Packag. Manuf. Technol..

[77] Kingsley N., Wang G.A., Papapolymerou J. 14 GHz microstrip MEMS phase shifters on flexible, organic substrate. Proceedings of the 35th European Microwave Conference (EuMC).

[78] Kingsley N., Papapolymerou J. (2006). Organic “Wafer-Scale” Packaged Miniature 4-bit RF MEMS Phase Shifter. IEEE Trans. Microw. Theory Tech..

[79] Wang G., Bairavasubramanian R., Pan B., Papapolymerou J. (2011). Radiofrequency MEMS-enabled polarisation-reconfigurable antenna arrays on multilayer liquid crystal polymer. IET Microw. Antennas Propag..

[80] Kingsley N., Ponchak G.E., Papapolymerou J. (2008). Reconfigurable RF MEMS phased array antenna integrated within a liquid crystal polymer (LCP) system-on-package. IEEE Trans. Antennas Propag..

[81] Lin C., Lixin X., Qi H. (2015). Radiation characteristics of a flexible micro-electro-mechanical system antenna on a liquid crystal polymer substrate. Mater. Res. Innov..

[82] Wright M.D., Baron W., Miller J., Tuss J., Zeppettella D., Ali M. (2018). MEMS Reconfigurable Broadband Patch Antenna for Conformal Applications. IEEE Trans. Antennas Propag..

[83] Kingsley N., Anagnostou D.E., Tentzeris M., Papapolymerou J. (2007). RF MEMS Sequentially Reconfigurable Sierpinski Antenna on a Flexible Organic Substrate with Novel DC-Biasing Technique. J. Microelectromech. Syst..

[84] Nadh B.P., Madhav B.T.P., Kumar M.S., Kumar T.A., Rao M.V., Reddy S.S.M. (2022). MEMS-based reconfigurable and flexible antenna for body-centric wearable applications. J. Electromagn. Waves Appl..

[85] Sailaja B.V.S., Naik K.K. (2021). Design and analysis of compact antenna with cascaded elliptical patch for reconfigurability using RF switches at satellite applications. Eur. Phys. J. Plus.

[86] Sailaja B.V.S., Naik K.K. (2021). Design and analysis of reconfigurable fractal antenna with RF-switches on a flexible substrate for X-band applications. Analog. Integr. Circuits Process..

[87] Lin C., Lixin X., Qi H., Tao D. Bending properties of a flexible micro-electro-mechanical system array antenna. Proceedings of the 2016 IEEE International Conference on Microwave and Millimeter Wave Technology (ICMMT).

[88] Cong L., Xu L.X., Li J.H., Wang T., Han Q. (2017). The conical conformal MEMS quasi-end-fire array antenna. Mod. Phys. Lett. B.

[89] Chung D.J., Bhattacharya S., Ponchak G.E., Papapolymerou J. A ‘stitched’ flexible light weight multilayer 16 × 16 antenna array on LCP. Proceedings of the 2009 European Microwave Conference (EuMC).

[90] Lin C., Lixin X., Qi H., Tao D. (2015). Process design and simulation of a flexible MEMS array antenna. Proceedings of the 2015 2nd International Workshop on Materials Engineering and Computer Sciences.

[91] Pestana T.G., Pinto R.M.R., Dias R.A., Martins M., Chu V., Gaspar J., Conde J.P. (2020). Fabrication and characterization of thin-film silicon resonators on 10 mu m-thick polyimide substrates. J. Micromech. Microeng..

[92] Petroni S., Maruccio G., Guido F., Amato M., Campa A., Passaseo A., Todaro M.T., De Vittorio M. (2012). Flexible piezoelectric cantilevers fabricated on polyimide substrate. Microelectron. Eng..

[93] Kang Y.R., Kang S.C., Paek K.K., Kim Y.K., Kim S.W., Ju B.K. (2005). Air-gap type film bulk acoustic resonator using flexible thin substrate. Sens. Actuator A Phys..

[94] Jiang Y., Zhang M.L., Duan X.X., Zhang H., Pang W. (2017). A flexible, gigahertz, and free-standing thin film piezoelectric MEMS resonator with high figure of merit. Appl. Phys. Lett..

[95] Courreges S., Morcillo C.A.D., Bhattacharya S., Papapolymerou J. (2010). Reduced-size multilayer X-band filters with stacked resonators on a flexible organic substrate. IET Microw. Antennas Propag..

[96] Yu L., Jin H., Hu N., Dong S. (2016). Flexible film bulk acoustic resonators and filter-like structure made directly on polymer substrates. Integr. Ferroelectr..

[97] Gao C., Zhang M., Jiang Y. (2019). FlexMEMS-enabled hetero-integration for monolithic FBAR-above-IC oscillators. Nanotechnol. Precis. Eng..

[98] Jiang Y., Zhao Y., Zhang L., Liu B.H., Li Q.N., Zhang M.L., Pang W. (2018). Flexible Film Bulk Acoustic Wave Filters toward Radiofrequency Wireless Communication. Small.

[99] Sun X., Zhang M.L., Gao C.H., Ning Y., Yuan Y., Pang W. (2019). Flexible lamb wave resonators with high figure of merit. Appl. Phys. Lett..

[100] Zhang L., Gao C.H., Jiang Y., Liu B.H., Zhang M.L., Zhang H.X., Li Q.N., Chen X.J., Pang W. (2019). Highly Bendable Piezoelectric Resonators for Flexible Radio-Frequency Electronics. Adv. Electron. Mater..

[101] Wright R.V., Hakemi G., Kirby P.B. (2011). Integration of thin film bulk acoustic resonators onto flexible liquid crystal polymer substrates. Microelectron. Eng..

[102] Han L., Wang R., Chen L.J. (2021). Bending characteristics of radio frequency microelectromechanical system low-pass filter based on flexible substrate. Electron. Lett..

[103] Yang Z., Kraman M.D., Zheng Z., Zhao H., Li X. (2020). Monolithic Heterogeneous Integration of 3D Radio Frequency LC Elements by Self In olled:p Membrane Nanotechnology. Adv. Funct. Mater..

[104] Coutts G.M., Mansour R.R., Chaudhuri S.K. (2007). A MEMS-tunable frequency-selective surface monolithically integrated on a flexible substrate. Proceedings of the IEEE/MTT-S International Microwave Symposium.

[105] Liu C. (2007). Recent developments in polymer MEMS. Adv. Mater..

[106] Thompson D.C., Tantot O., Jallageas H., Ponchak G.E., Tentzeris M.M., Papapolymerou J. (2004). Characterization of liquid crystal polymer (LCP) material and transmission lines on LCP substrates from 30 to 110 GHz. IEEE Trans. Microw. Theory Tech..

[107] Altunyurt N., Swaminathan M., Sundaram V., White G. Conformal Antennas on Liquid Crystalline Polymer Substrates for Consumer Applications. Proceedings of the 2007 Asia-Pacific Microwave Conference.

[108] Ha D., de Vries W.N., John S.W.M., Irazoqui P.P., Chappell W.J. (2012). Polymer-based miniature flexible capacitive pressure sensor for intraocular pressure (IOP) monitoring inside a mouse eye. Biomed. Microdevices.

[109] Jiang X., Wang C.H., Liu W. (2015). A Laser-Assisted Bonding Method Using a Liquid Crystal Polymer Film for MEMS and Sensor Packaging. IEEE Trans. Compon. Packag. Manuf. Technol..

[110] Rihani R., Tasnim N., Javed M., Usoro J.O., D’Souza T.M., Ware T.H., Pancrazio J.J. (2021). Liquid Crystalline Polymers: Opportunities to Shape Neural Interfaces. Neuromodulation Technol. Neural Interface.

[111] Kottapalli A.G.P., Asadnia M., Miao J.M., Barbastathis G., Triantafyllou M.S. (2012). A flexible liquid crystal polymer MEMS pressure sensor array for fish-like underwater sensing. Smart Mater. Struct..

[112] Palasagaram J.N., Ramadoss R. (2006). MEMS-capacitive pressure sensor fabricated using printed-circuit-processing techniques. IEEE Sens. J..

[113] Kottapalli A.G.P., Tan C.W., Olfatnia M., Miao J.M., Barbastathis G., Triantafyllou M. (2011). A liquid crystal polymer membrane MEMS sensor for flow rate and flow direction sensing applications. J. Micromech. Microeng..

[114] Kottapalli A.G.P., Asadnia M., Miao J.M., Tan C.W., Barbastathis G., Triantafyllou M. (2012). Polymer MEMS pressure sensor arrays for fish-like underwater sensing applications. Micro Nano Lett..

[115] Altunyurt N., Rieske R., Swaminathan M., Sundaram V. (2009). Conformal Antennas on Liquid Crystalline Polymer Based Rigid-Flex Substrates Integrated with the Front-End Module. IEEE Trans. Adv. Packag..

[116] Hu N.N., He X.L., Bian X.L., Chen G.H., Dong S.R., Luo J.K. Novel flexible FBAR on PET substrate. Proceedings of the 2014 IEEE International Conference on Electron Devices and Solid-State Circuits.

[117] Yan Z., Zhang F., Wang J., Liu F., Guo X., Nan K., Lin Q., Gao M., Xiao D., Shi Y. (2016). Controlled Mechanical Buckling for Origami-Inspired Construction of 3D Microstructures in Advanced Materials. Adv. Funct. Mater..

[118] Rivadeneyra A., Fernandez-Salmeron J., Agudo-Acemel M., Palma A.J., Capitan-Vallvey L.F., Lopez-Villanueva J.A. (2015). Cantilever Fabrication by a Printing and Bonding Process. J. MicroelectroMech. Syst..

[119] Ramadoss R., Lee S., Bright V.M., Lee Y.C., Gupta K.C. Polyimide film based RF MEMS capacitive switches. Proceedings of the IEEE MTT-S International Microwave Symposium.

[120] Su L.J., Huang X., Guo W., Wu H. (2020). A Flexible Microwave Sensor Based on Complementary Spiral Resonator for Material Dielectric Characterization. IEEE Sens. J..

[121] Mahmood M.S., Celik-Butler Z., Butler D.P. (2017). Design, fabrication and characterization of flexible MEMS accelerometer using multi-Level UV-LIGA. Sens. Actuators A Phys..

[122] Ramadoss R., Lee S., Lee Y.C., Bright V.M., Gupta K.C. (2005). Flexible polyimide film based high isolation RE MEMS switches fabricated using printed circuit processing techniques. Proceedings of the 18th IEEE International Conference on Micro Electro Mechanical Systems (MEMS).

[123] Mahmood M.S. Design and Fabrication of Self-Packaged, Flexible MEMS Accelerometer. Proceedings of the 2015 IEEE SENSORS.

[124] Chen G., Zhao X., Wang X., Jin H., Li S., Dong S., Flewitt A.J., Milne W.I., Luo J.K. (2015). Film bulk acoustic resonators integrated on arbitrary substrates using a polymer support layer. Sci. Rep..

[125] Frazier A.B. (1995). Recent applications of polyimide to micromachining technology. IEEE Trans. Ind. Electron..

[126] Shi Y., Wang C., Yin Y., Li Y., Xing Y., Song J. (2019). Functional Soft Composites as Thermal Protecting Substrates for Wearable Electronics. Adv. Funct. Mater..

[127] Yin Y., Li M., Li Y., Song J. (2020). Skin pain sensation of epidermal electronic device/skin system considering non-Fourier heat conduction. J. Mech. Phys. Solids.

[128] Sundani H., Devabhaktuni V., Melkonian C., Alam M. (2013). Design and fabrication of a micromachined free-floating membrane on a flexible substrate. J. Micro/Nanolithography MEMS MOEMS.

[129] Wang C., Linghu C., Nie S., Li C., Song J. (2020). Programmable and scalable transfer printing with high reliability and efficiency for flexible inorganic electronics. Sci. Adv..

[130] Saeidpourazar R., Li R., Li Y., Sangid M.D., Lu C., Huang Y., Rogers J.A., Ferreira P.M. (2012). Laser-Driven Micro Transfer Placement of Prefabricated Microstructures. J. Microelectromech. Syst..

[131] Li R., Li Y., Lü C., Song J., Saeidpouraza R., Fang B., Zhong Y., Ferreira P.M., Rogers J.A., Huang Y. (2012). Thermo-mechanical modeling of laser-driven non-contact transfer printing: Two-dimensional analysis. Soft Matter.

[132] Chen H., Feng X., Chen Y. (2013). Directionally controlled transfer printing using micropatterned stamps. Appl. Phys. Lett..

[133] Gao Y., Li Y., Li R., Song J. (2017). 1-An accurate thermo-mechanical model for laser-driven micro-transfer printing. J. Appl. Mech..

[134] Luo H., Wang C., Linghu C., Yu K., Wang C., Song J. (2019). Laser-Driven Programmable Non-Contact Transfer Printing of Objects onto Arbitrary Receivers via an Active Elastomeric Micro-Structured Stamp. Natl. Sci. Rev..

[135] Sirringhaus H., Kawase T., Friend R.H., Shimoda T., Inbasekaran M., Wu W., Woo E.P. (2000). High-resolution inkjet printing of all-polymer transistor circuits. Science.

[136] Yin Z.P., Huang Y.A., Bu N.B., Wang X.M., Xiong Y.L. (2010). Inkjet printing for flexible electronics: Materials, processes and equipments. Chin. Sci. Bull..

[137] Hester J.G., Kim S., Bito J., Le T.R., Kimionis J., Revier D., Saintsing C., Su W.J., Tehrani B., Traille A. (2015). Additively Manufactured Nanotechnology and Origami-Enabled Flexible Microwave Electronics. Proc. IEEE.

[138] Adams J.J., Duoss E.B., Malkowski T.F., Motala M.J., Bok Yeop A., Nuzzo R.G., Bernhard J.T., Lewis J.A. (2011). Conformal printing of electrically small antennas on three-dimensional surfaces. Adv. Mater..

[139] Cheng X., Zhang Y.H. (2019). Micro/Nanoscale 3D Assembly by Rolling, Folding, Curving, and Buckling Approaches. Adv. Mater..

[140] Zhang Y., Zhang F., Yan Z., Ma Q., Li X., Huang y.-s., Rogers J. (2017). Printing, folding and assembly methods for forming 3D mesostructures in advanced materials. Nat. Rev. Mater..

[141] Kim B.H., Li K., Kim J.-T., Park Y., Jang H., Wang X., Xie Z., Won S.M., Yoon H.-J., Lee G. (2021). Three-dimensional electronic microfliers inspired by wind-dispersed seeds. Nature.

[142] Bai K., Cheng X., Xue Z., Honglie S., Sang L., Zhang F., Liu F., Luo X., Huang W., Huang Y.-S. (2020). Geometrically reconfigurable 3D mesostructures and electromagnetic devices through a rational bottom-up design strategy. Sci. Adv..

[143] Liu F., Cheng X., Zhang F., Chen Y., Song H.L., Huang Y.G., Zhang Y.H. (2019). Design and Assembly of Reconfigurable 3D Radio-Frequency Antennas Based on Mechanically Triggered Switches. Adv. Electron. Mater..

[144] Liu F., Chen Y., Song H.L., Zhang F., Fan Z.C., Liu Y., Feng X., Rogers J.A., Huang Y.G., Zhang Y.H. (2019). High Performance, Tunable Electrically Small Antennas through Mechanically Guided 3D Assembly. Small.

[145] Fu H., Nan K., Bai W., Huang W., Bai K., Lu L., Zhou C., Liu Y., Liu F., Wang J. (2018). Morphable 3D mesostructures and microelectronic devices by multistable buckling mechanics. Nat. Mater..

[146] Jang K.-I., Li K., Chung H.U., Xu S., Jung H.N., Yang Y., Kwak J.W., Jung H.H., Song J., Yang C. (2017). Self-assembled three dimensional network designs for soft electronics. Nat. Commun..

[147] Xu S., Yan Z., Jang K.I., Huang W., Fu H.R., Kim J., Wei Z., Flavin M., McCracken J., Wang R. (2015). Assembly of micro/nanomaterials into complex, three-dimensional architectures by compressive buckling. Science.

[148] Yan Z., Zhang F., Liu F., Han M.D., Ou D.P., Liu Y.H., Lin Q., Guo X.L., Fu H.R., Xie Z.Q. (2016). Mechanical assembly of complex, 3D mesostructures from releasable multilayers of advanced materials. Sci. Adv..

[149] Yin Y.F., Zhao Z., Li Y.H. (2021). Theoretical and experimental research on anisotropic and nonlinear mechanics of periodic network materials. J. Mech. Phys. Solids.

[150] Zhang Y.H., Yan Z., Nan K.W., Xiao D.Q., Liu Y.H., Luan H.W., Fu H.R., Wang X.Z., Yang Q.L., Wang J.C. (2015). A mechanically driven form of Kirigami as a route to 3D mesostructures in micro/nanomembranes. Proc. Natl. Acad. Sci. USA.

[151] Ling Y., Zhuang X.T., Xu Z., Xie Y.C., Zhu X.Y., Xu Y.D., Sun B.H., Lin J., Zhang Y.H., Yan Z. (2018). Mechanically Assembled, Three-Dimensional Hierarchical Structures of Cellular Graphene with Programmed Geometries and Outstanding Electromechanical Properties. ACS Nano.

[152] Lee W., Liu Y., Lee Y., Sharma B.K., Shinde S.M., Kim S.D., Nan K., Yan Z., Han M.D., Huang Y.G. (2018). Two-dimensional materials in functional three-dimensional architectures with applications in photodetection and imaging. Nat. Commun..

[153] Molina-Lopez F., Briand D., Rooij N.F.D. Large arrays of inkjet-printed MEMS microbridges on foil. Proceedings of the 2014 IEEE 27th International Conference on Micro Electro Mechanical Systems (MEMS).

